# Delayed Sowing Reduced *Verticillium* Wilt by Altering Soil Temperature and Humidity to Enhance Beneficial Rhizosphere Bacteria of Sunflower

**DOI:** 10.3390/microorganisms12122416

**Published:** 2024-11-25

**Authors:** Jianfeng Yang, Shuo Jia, Tie Li, Jian Zhang, Yuanyuan Zhang, Jianjun Hao, Jun Zhao

**Affiliations:** 1 College of Horticulture and Plant Protection, Inner Mongolia Agricultural University, Hohhot 010019, China; jianfengyang89@126.com (J.Y.);; 2 Hinngan League Institute of Agricultural and Husbandry Sciences, Ulanhot 134000, China; 3 Ministry of Agriculture and Rural Affairs, Institute of Grassland Research of CAAS, Hohhot 010010, China; 4 School of Food and Agriculture, University of Maine, Orono, ME 04469, USA

**Keywords:** sowing date, disease severity, SynComs, antagonistic effect

## Abstract

Sunflower *Verticillium* Wilt (SVW) caused by *Verticillium dahliae* is a significant threat to sunflower production in China. This soilborne disease is difficult to control. It has been observed that delayed sowing reduces the severity of SVW on different varieties and across various locations. Soil was collected from multiple locations with different sowing dates to understand the underlying biological mechanisms driving this phenomenon. The soil bacterial community was characterized through 16S rRNA gene amplicon sequencing performed on the Illumina MiSeq platform, followed by comprehensive bioinformatics analysis. Microsclerotia numbers in soil were detected using both NP-10 selective medium and quantitative polymerase chain reaction (qPCR). By delaying the sowing date, the number of microsclerotia in soil and the biomass of *V. dahliae* colonized inside sunflower roots were reduced during the early developmental stages (V2–V6) of sunflowers. Amplicon sequencing revealed an increased abundance of bacterial genera, such as *Pseudomonas*, *Azoarcus*, and *Bacillus* in soil samples collected from delayed sowing plots. Five bacterial strains isolated from the delayed sowing plot exhibited strong antagonistic effects against *V. dahliae*. The result of the pot experiments indicated that supplying two different synthetic communities (SynComs) in the pot did increase the control efficiencies on SVW by 19.08% and 37.82% separately. Additionally, soil temperature and humidity across different sowing dates were also monitored, and a significant correlation between disease severity and environmental factors was observed. In conclusion, delayed sowing appears to decrease microsclerotia levels by recruiting beneficial rhizosphere bacteria, thereby reducing the severity of SVW.

## 1. Introduction

Sunflower *Verticillium* Wilt (SVW) is caused by the soilborne fungus *Verticillium dahliae* Kleb. This fungus produces microsclerotia, which are long-term survival structures [1]. Microsclerotia can germinate when exposed to favorable environmental conditions and host root exudates [2,3]. Once germinated, the hyphae infect and colonize host roots, eventually penetrating the root vascular system. The fungus spreads systemically through the plant via mycelia and conidia [4,5].

Symptoms of SVW are typically observed in the field during the sunflower flowering season and manifest as chlorosis and premature plant death. This disease is monocyclic, with microsclerotia produced in the above-ground and root-dead tissue [3]. As plant debris decays, microsclerotia are released into the soil, where they spread through cultivation practices and serve as the resource of primary infection.

Controlling VW is particularly challenging due to its broad host range and the formation of microsclerotia, which can persist in soil for over a decade [6]. Effective management of SVW requires an integrated approach, including planting resistant varieties [7], rotating with non-host crops [8], and utilizing disease-suppressive green manures [9]. Additionally, adjusting sowing dates can help reduce disease occurrence and its severity. This technique has been successfully employed to control various soilborne diseases, such as *Fusarium* wilt of chickpea, potyviral disease in tomato, and White Rust in Indian mustard [10,11,12]. For instance, Nehl and Anderson [13] found that delaying the sowing date was the most effective strategy to mitigate *Fusarium* wilt on cotton, and similar results were reported that delaying the sowing date decreased the disease index of Wheat Sharp Eyespot [14]. However, the biological mechanisms underlying these effects remain elusive.

Generally, the amount of the pathogen in the soil is highly correlated with the disease severity [15]. Microsclerotial density in the soil can serve as a crucial determinant of disease severity in subsequent seasons [16,17]. However, other factors such as pathotype, temperature, and irrigation frequency can also influence the severity of VW [18]. The composition of rhizosphere microbial communities is strongly influenced by plant species [19]. Plant-associated microbial communities are essential for plant growth and play a pivotal role in disease suppression. Several studies have demonstrated that rhizosphere microbes or endophytic microbiomes can be manipulated by phytopathogenic fungi, thereby facilitating pathogen infection [20,21]. While pathogen attack can alter the community composition and function of rhizosphere microorganisms, it remains unclear whether these changes are a direct consequence of infection or actively driven by the host plant.

In the natural environment, plant development is frequently affected by both biotic and abiotic stresses. The responses of plants to these stresses have been extensively studied and well-characterized, particularly in mutualistic systems involving individual plant species exposed to specific stress conditions [22,23]. Biological stresses, such as pathogens, insects, and nematodes, can significantly impact plant development [24]. Abiotic stresses, such as temperature, humidity, light, soil structure, and aeration, also influence the resistance levels of plants [25,26,27]. Various biotic and abiotic factors greatly influence the occurrence, development, and epidemiology of plant diseases. Some factors can exacerbate disease severity [28], some directly cause non-infectious diseases [25,29], and some can either promote or attenuate disease severity under different environmental conditions [22,23].

Plant microbiome studies have generated extensive sequencing data and insights into the diversity and abundance of microbial communities across various plant habitats, including the rhizosphere, phyllosphere, seeds, and germ layers. Numerous studies have highlighted how these microbial communities impact specific plant phenotypes, such as plant growth, development, and pathogen infection [30,31,32,33,34,35,36]. However, the causal relationship between plant resistance level and microbiota structure and function at the molecular and biochemical levels remains poorly understood.

Bacteria and fungi play essential roles in regulating the biological, chemical, and physical processes in terrestrial ecosystems [37,38], and influence soil properties, including organic matter decomposition, toxin degradation, and biogeochemical cycling [39]. Bacterial communities in soils are typically richer and more diverse than fungal communities [40] and contribute significantly to soil health and agricultural productivity [41]. Additionally, bacteria and fungi in the soil promote plant growth by competing for ecological niches, thereby inhibiting and restricting pathogen growth and colonization in the rhizosphere. This competition significantly influences the occurrence and development of soilborne diseases [42,43,44].

The occurrence of soilborne diseases often coincides with structural imbalances in the microbial communities within the rhizosphere of host plants, alongside microecological deterioration, which exacerbates the prevalence of soilborne diseases [45,46]. Recent research has confirmed that the diversity of soil microbial communities is closely correlated with the resistance levels of host plants. Soil microbial communities can enhance plant resistance, effectively acting as a “plant umbrella” by limiting pathogen invasion [47,48]. For example, some antibiotic compounds produced by *Streptomyces* can directly reduce pathogenic bacterial population [49]. Disease-resistant varieties of common legumes, which contain antifungal compounds and promote plant growth, are significantly enriched with beneficial endophytes, such as *Pseudomonas*, *Bacillus*, *Solanaceae*, and *Fibrophagaceae* [50]. Additionally, the severity of potato common scab and the abundance of pathogenic *streptomycetes* are positively correlated with bacterial genera, such as *Metarhizium*, *Oligotrophomonas*, and *Agrobacterium* [51]. It has been demonstrated that the suppression of soilborne diseases is closely related to the diversity and abundance of soil-beneficial microbial communities [52].

SVW caused by *V. dahliae* is a significant threat to sunflower production in China. Recently, due to continuous cropping, the severity of SVW in most sunflower-producing areas has intensified. Experiments in delaying the sowing date were performed with different varieties across multiple locations, consistently showing that disease severity decreased dramatically with delayed sowing date. Based on this phenomenon, we aim to investigate whether there is a significant correlation between the changes in environmental factors following delayed sowing, and if this is the driving force for the decrease in the severity of SVW. Additionally, we try to confirm whether delayed sowing also facilitates the transition of the composition of the microbiome in the rhizosphere of sunflower roots. The results of this study will lay a cornerstone for creating an effective agronomic technique to control VW in the future.

## 2. Materials and Methods

Field information and sampling strategy. Fields in a sunflower-growing region of Inner Mongolia and Gansu Province in 2017–2019 were selected for the investigation. Three different field locations were used in the study (Table 1). We set up the plots in the main sunflower-producing regions where sunflowers have been continuously cultivated for at least three years and SVW occurrence is severe. A farmland plot was uniformly divided into 25 subplots (a subplot consisting often plants in three rows), and the subplot area was around 40 m^2^ to ensure that each plot planted at least 80 plants, with a row spacing of 50 cm and 90 cm line spacing. Sunflower seeds were sown at five different sowing times, with each sowing time consisting of five randomly distributed subplots. The starting date was variable due to the local climate conditions. Sunflower varieties were selected according to the cumulative temperature in the corresponding region (Table 1), and resistant and susceptible varieties were selected based on the resistant level identification result in the field [53,54,55].

Soil samples were collected at four distinct growth stages of sunflowers: V2 (4th leaf stage), V6 (8th leaf stage), R1 (early budding period), and R5 (mid-blooming period), to assess the dynamic changes in the biomass of *V. dahliae* in the rhizosphere soil across different stages of plant development. In each subplot, five points were marked in a Z-shaped pattern. The four plants closest to each marked point were selected as sampling plants. Specifically, at each of the sampling stages, soil samples (100 g) were collected using a soil auger at a distance of 10 cm from the stem of the plant. The samples included both the root and its surrounding soil. The samples were stored at low temperatures and transported back to the laboratory [56]. Soil attached to roots was gently shaken off in an ultra-clean bench, and the soil adhering to the roots was defined as the rhizosphere soil [57]. Rhizosphere soil was filtered with a 100 μm nylon mesh and kept in a 50 mL tube. Replicates were made with 500 μL 20% rhizosphere soil and 500 μL 80% glycerol, then frozen in liquid nitrogen. Root tissues were rinsed with sterile 10 mM PBS solution, snap-frozen in liquid nitrogen, and stored at −80 °C. Rhizosphere soil samples were collected to assess the abundance of pathogens in the soil environment, while root tissue samples were obtained to quantify the presence of pathogens colonizing the plant. Additionally, rhizosphere soil from location 3 was also sequenced using the Illumina MiSeq platform to characterize the composition of the microbial (bacterial) community across different plots with different sowing dates.

Disease assessments in fields. The disease grade of SVW was investigated during the flowering period (R5–R6) at different sowing dates in field experiments (2017–2019). Within each experimental subplot, disease severity was assessed and recorded for 50 individual plants. The number of plants showing typical symptoms was recorded, and the disease grade was determined based on the criteria described by Flood [58]. Disease grades (0 to 4) were classified based on the percentage of chlorosis areas relative to total leave area: 0 = no symptoms, 1 ≤ 25%, 2 = wilting and stunting ≥ 25%, 3 wilting and stunting ≥ 50%, 4 = wilting and stunting ≥ 75%. The disease index was calculated using the following formula:

Disease index = [((0 × N0) + (1 × N1) + (2 × N2) + (3 × N3) + (4 × N4))/(N1 + N2 + N3 + N4)] × 100,

where N = the number of plants with the respective disease grade.

Measurement of yield. At physiological maturity, fifty random sunflower heads within each subplot were harvested, dried, and threshed to determine the total seed yield per subplot. Thousand-seed weight was measured by randomly selecting and weighing 1000 seeds from the harvested yield, ensuring uniform moisture content before weighing to standardize measurements. Measurements were taken from five subplots, and the average was calculated to represent the mean yield and thousand-seed weight for each subplot.

Measurement of soil environmental factors. Soil physicochemical properties were measured using the Soil Rapid Measurement Platform (SRMP: JXBS-3001-SCY, Weihai Jingxun Changtong Electronic Technology Co., Ltd., Weihai, China). During the sunflower sowing season in 2019, real-time monitoring of soil temperature, humidity, and pH was conducted with SRMP. Sunflowers were planted across 25 subplots at different sowing dates, with measurements of soil properties in five subplots of each sowing date. Briefly, a soil sensor was inserted 15 cm into the soil, and readings were recorded after a 15 min equilibration period. Measurements were repeated multiple times to ensure accuracy.

Pot experiments with different sowing dates. To mitigate potential confounding factors in field experiments, such as hail, strong winds, and adverse impacts of extreme weather, we conducted pot experiments in an open greenhouse at the university. While the greenhouse was protected from rainfall, it did not maintain stable temperature and humidity conditions (the temperature varied according to local weather fluctuations, ranging from 16.8 °C to 39.3 °C, while relative humidity ranged from 22.5% to 76.5%). Pots with a 35 cm diameter were filled with sterilized soil and inoculated with *V. dahliae* strain GN3 using the soil inoculation method [59]. Briefly, a potato dextrose agar (PDA) plug with *V. dahliae* was transferred into Erlenmeyer flasks containing wheat bran medium and was kept in the dark after inoculation. A total of 15 days later, the culture was dried and pulverized in a sterilized mortar, then mixed with sterilized soil with a ratio of 2.0%. The conidial concentration in the soil reached 1 × 10^7^ conidia per gram. Fifteen pots were prepared for each sowing date, with six sunflower seeds planted in each pot. The first sowing date began on 1 May, followed by other sowing dates with 10-day intervals. Pots were kept in an open greenhouse, and disease assessments were conducted using standard disease scales 45 days after sowing [58]. The disease grade and the formula for calculating the disease index are the same as in the field experiment design.

The soil sampling (200 g/plant) and pretreatment methods for rhizosphere soil were consistent with those used in the field experiment. However, in the pot experiment, rhizosphere soil collected from three sunflowers planted in one pot was mixed to represent one sample for each pot. Samples from five pots were then combined to represent each sowing date treatment. Similarly, at the time of rhizosphere soil collection, sunflower root tissues (1 g/plant) were quickly excised and flash-frozen in liquid nitrogen, then stored in an ultra-low temperature freezer. The samples were stored at low temperatures and transported back to the laboratory [56]. Soil attached to roots was gently shaken off in an ultra-clean bench, and the soil adhering to the roots was defined as the rhizosphere soil [57]. The rhizosphere soil was sieved through a 100 µm nylon mesh and collected in a 50 mL centrifuge tube. The resulting pellet was resuspended in 1 mL of phosphate buffer and transferred to a 2 mL microcentrifuge tube. An equal volume of 80% glycerol stock solution was added, and the mixture was flash-frozen in liquid nitrogen. Roots were rinsed with sterile 10 mM PBS solution, snap-frozen in liquid nitrogen, and stored at −80 °C.

DNA/RNA extraction. Total DNA from rhizosphere soil was extracted using a PowerSoil DNA Isolation Kit (Qiagen, Hilden, Germany) as per the manufacturer’s instruction. DNA quality was assessed on 1% agarose gel, and concentrations were measured with a NanoDrop 2000 spectrophotometer (Thermo Scientific, Wilmington, DC, USA). Total RNA was extracted from the roots using TRIzol reagent (Invitrogen, Waltham, MA, USA) [60]. All samples were ground to a fine powder using a mortar and pestle in liquid nitrogen. The total RNA was eluted in RNase-free water and stored at −80 °C until further use. Each experiment consisted of five biological replicates. Reverse transcription was then performed to obtain cDNA using the SuperScript III reverse transcriptase (Invitrogen, Carlsbad, CA, USA).

Microsclerotia number and pathogen biomass detection. Our previous study developed a rapid method for quantitatively assessing the number of Microsclerotia in soil samples [61]. Briefly, selective medium NP-10 enabled the efficient isolation and cultivation of *V. dahliae* from the soil. Additionally, the soil sample was uniformly spread across a culture plate based on aerodynamic principles, facilitating accurate enumeration of the colonies. Meanwhile, quantitative real-time PCR (qRT-PCR) was performed with specific primers *VertBt*-F/R (5′-CATCAGTCTCTCTGTTTATACCA ACG-3′ and 5′- CGATGCGAGCTGTAACTACTACGCAA-3′) to detect biomass of *V. dahliae* colonized inside the sunflower roots. RNA was reverse transcribed using SuperScript III reverse transcriptase (Invitrogen) and oligo(dT) primers to obtain cDNA. qRT-PCR was performed using SYBR^®^ Premix Ex Taq™ II (Tli RNaseH Plus) according to the manufacturer’s protocol (Takara, Kusatsu, Shiga, Japan). The qRT-PCR procedure included an initial denaturation step at 95 °C for 10 min, followed by 40 cycles of 95 °C for 15 s and 60 °C for 1 min. The sunflower Actin gene (*HaActin*) was used as an endogenous control to normalize the expression levels of target genes. Relative transcript levels were calculated using the 2^−∆∆CT^ method for three independent biological replicates [62].

Amplicon sequencing. We collected rhizosphere soil samples from the aforementioned four stages (Table 2) and conducted microbial (bacterial) diversity analyses. To assess differences in microbial (bacterial) diversity in soil collected from different sowing date plots, we sequenced rhizosphere soil samples from V2 and V6 developmental stages in the first (S1) and fifth (S5) sowing date plots at location 3 (Table 1). Purified amplicons were pooled in equimolar and paired-end sequenced on an Illumina MiSeq PE300 platform (Illumina, San Diego, CA, USA) according to the standard protocols by Majorbio Bio-Pharm Technology Co., Ltd. (Shanghai, China). The raw reads were deposited in the NCBI Sequence Read Archive (SRA) database. The raw 16S rRNA gene sequencing reads were demultiplexed and quality-filtered using FASTP version 0.20.0, and merged using FLASH version 1.2.7 under the following criteria: (i) reads were truncated at any site with an average quality score < 20 over a 50 bp sliding window, and discarded if shorter than 50 bp or containing ambiguous characters; (ii) overlapping sequences longer than 10 bp were assembled with a maximum mismatch ratio of 0.2; (iii) exact barcode matching and primer alignment with up to 2 mismatches were used for sequence distinction [63,64].

Operational taxonomic units (OTUs) with a 97% similarity cutoff [65,66] were clustered using UPARSE version 7.1, with chimeric sequences identified and removed. The taxonomy of each OTU representative sequence was analyzed using RDP Classifier version 2.2 against the 16S rRNA database (e.g., Silva v138), using a confidence threshold of 0.7 [67].

Microbe isolation and identification from soil samples. The rhizosphere soil samples collected from different sowing date plots were used for microbial isolation. The pre-processed samples were resuspended in PBS buffer (0.1 M phosphate buffer, 0.15% Tween 80, pH 7.0) and subjected to microbial isolation using the limiting dilution method [68]. A 100 μL dilution was placed on five different culture media, including 1/10 strength tryptic soy agar (TSA), tap water yeast extract (TWYE), tryptone yeast extract glucose (TYG), Luria-Bertani (LB), and nutrient agar (NA) (Supplementary Data S1) for bacterial isolation. Plates were incubated at 28 °C. Colonies were selected based on size, color, and morphology, then transferred to 96-well plates cultured in LB for five days. DNA was extracted using lysis buffer (0.25% SDS, 0.05 N NaOH) and incubated for 10 min at 95 °C.

The DNA extracted from the 96-well plates culture was submitted for PCR to identify bacteria species [69]. The 16S rRNA gene was amplified using the forward primer 505F (5′GTGCCAGCMGCCGCGGTAA-3′) and reverse primer 806R (5′-GGACTACHVGGGTWTCTAAT3′). The PCR procedure was as follows: denaturation at 95° for 4 min, followed by 25 cycles of 95 °C for 30 s, 55 °C for 30 s, and 72 °C for 60 s, with a final elongation at 72 °C for 10 min.

Different SynComs generation and antagonist effects. Based on previous research, we isolated 610 bacterial strains that were significantly enriched in the rhizosphere soil of late-sown sunflowers. To evaluate whether these specific strains possess disease-suppressive capabilities, we selected five bacterial strains exhibiting antagonistic activity against *Verticillium dahliae* (determined via plate confrontation assays) and three non-antagonistic strains to construct a SynCom. We designed a controlled pot experiment to assess the disease suppression potential of different SynComs. Detailed experimental methods are provided as follows: the germ-free sunflower seedlings were transferred to a plant growth incubator with a 16/8 h day/night cycle. Six sunflower seedling pots (biological replicates) were prepared for each treatment, with 5 plants in each pot. To verify that the germ-free seedlings remained uncontaminated, soil from the rhizosphere of axenic control sunflowers was diluted to 10^−2^ concentrations, and 100 μL was plated in TSA plates. No colonies were observed, confirming no contamination of soil in a pot. All the bacteria used in the experiment were obtained from soil samples collected from the late sowing date (10 June), and they were more extensively enriched compared with bacteria isolated from soil samples of the first sowing date (1 May) (Table 3). Bacterial strains were propagated using the shake flask fermentation method in LB for three days at 28 °C. The bacterial broth was centrifuged at 4000× *g* for 8 min and resuspended in PBS, adjusted to OD600 of 0.2 (~10^7^ cfu/mL). The bacterial strains were mixed in equal ratios, forming four different SynCom: SynCom1 (5 strains of antagonistic bacteria), SynCom2 (3 strains of non-antagonistic strains bacteria), SynCom3 (SynCom1 + SynCom2), and control (Table 3). Each bacterial SynComs was adjusted to 1 × 10^8^ cfu/mL before being poured into the soil. The plants were pretreated twice with 200 mL of synthetic microbial communities (SynCom) solution at the V2 stage. The solution was poured near the basal stem of the sunflower at 48 h intervals.

Seedlings were inoculated with a conidial suspension of *V. dahliae* (GN3) (1 × 10^7^ conidia/mL) using root drenching after three days of pretreatment with different SynComs. The pathogen inoculation way was described by Larkin and Fravel [70]. Disease grade was recorded at 45 days of sunflower growth. In parallel, a growth-promoting trial was also conducted. After seedling growth for two weeks, different SynCom solutions were applied to the pots, with an equivalent volume of water as the control. Plant height was recorded after 45 days of growth.

### Statistical Analysis

All statistical analyses were performed using SAS 9.0, SPSS 22.0, and Excel 2017. Data were checked for normality using the Shapiro–Wilk test, and homogeneity of variance was assessed with Levene’s test. For comparison of means, analysis of variance (ANOVA) was conducted, followed by Tukey’s Multiple Comparisons Test (TMCT) at a significance level of *p* < 0.05 to determine statistical differences among treatments. Correlation analysis was performed to evaluate the relationships between soil temperature, moisture, and disease severity using Pearson’s correlation coefficient. All data are presented as mean ± standard error (SE) from three biological replicates unless otherwise specified. Graphs were generated using OriginPro 2021 and GraphPad Prism 8.4.3, and statistical significance was indicated by different letters.

## 3. Results

### 3.1. Delayed Sowing Date Reduced the Severity of SVW

**Figure 1:**
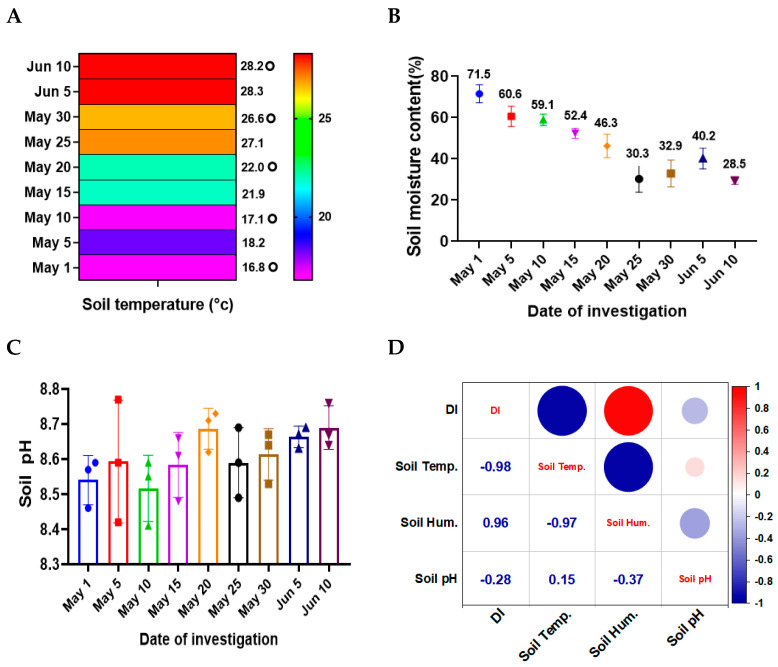
Correlation between soil temperature, moisture, and disease severity. (**A**) Soil temperature changes from first (1 May) to last sowing date (10 June); (**B**) Soil humidity changes with delayed sowing date. (**C**) Soil pH (acidity and alkalinity) changes with delayed sowing date; differences between treatment means were analyzed using one-way ANOVA, followed by Tukey’s HSD test (*p* < 0.05). Error bars represent standard deviation. The three points on each bar represent the mean values of three replicates. (**D**) Heatmap illustrating correlation between different environmental factors and disease index. DI, disease index; Temp., temperature; Hum., humidity. Correlation analysis was performed to evaluate the relationships between soil temperature, moisture, and disease severity using Pearson’s correlation coefficient. Graphs were generated using OriginPro 2021 and GraphPad Prism 8, and statistical significance is indicated by different letters. Numbers and circle sizes represent correlation coefficients and strength, separately. Negative sign represents a negative correlation.

We performed a delayed sowing experiment several times at different locations. In Minqin, Gansu Province in 2017, the disease index of the confectionary sunflower variety ‘LD5009’ decreased with delayed sowing. Specifically, the disease index dropped by 58% from the first sowing date (20 April) to the last sowing date (30 May). For the oil sunflower variety ‘T562’, the disease index decreased 27.86 at from the first sowing date (20 April) to 9.43 at the last sowing date (30 May), representing a 65.1% reduction. Additionally, the oil sunflower ‘T562’ showed much higher resistance to VW compared to the confectionary sunflower ‘LD 5009’ across different sowing dates (Figure 1A).

A similar trend was also observed in Wuyuan, Inner Mongolia, in 2018. The disease index for LD5009 decreased by 80.02%, while the resistance variety Keyang-7 showed an 82.54% reduction with a delayed sowing date. In Wulateqianqi, Inner Mongolia, in 2019, the disease index decreased by 55.71% for the susceptible variety LD5009 and by 83.48% for the resistant variety JK601 (Table 4).

Interestingly, in the pot experiment with artificial inoculation, delayed sowing also led to a decreased tendency on disease severity, regardless of the resistance level of the sunflower variety (Table 4).

All results obtained in both field and open greenhouse revealed that delayed sowing reduces the severity of SVW effectively.

### 3.2. Effect of Sowing Date on Sunflower Yield

After investigating the disease grade, the thousand kernel weight and the plot yield were also calculated in three different locations, Minqin, Wuyuan, and Wulateqianqi; the results of three different locations showed the same tendency (Table 5), and both the thousand kernel weight (TKW) and plot yield increased with delaying sowing date. In Wulateqianqi, Inner Mongolia, in 2019, the susceptible variety LD5009 had an average thousand-kernel weight (TKW) and subplot yield of 120.43 g and 5.67 kg, respectively, at the final sowing date increases of 28.91% and 50.79% compared to the initial sowing date. For the resistant variety JK601, the average TKW and subplot yield at the final sowing date were 146.21 g and 6.87 kg, showing increases of 41.89% and 54.04% relative to the initial sowing date. A similar trend was observed in field trials conducted in 2017 and 2018, indicating that delaying the sowing date significantly reduced disease incidence, thereby increasing yield.

### 3.3. Correlation Between Soil Temperature, Moisture, and Disease Severity of SVW

Through real-time measurements using the Soil Rapid Measurement Platform, we observed that all measured soil physical and chemical properties changed with the sowing date delayed. The most pronounced changes were observed in soil temperature and moisture. Notably, soil temperature was remarkably low at 16.8 °C on the first sowing date (1 May), and gradually increased to 28.2 °C at the last sowing date (10 June), displaying an upward trend over time (Figure 1A). However, intermediate measurements showed fluctuations in soil temperature, with both high and low extremes points, likely due to weather condition variations. Correspondingly, soil moisture content also exhibited a marked decline, decreasing from 71.5% at the first sowing date (1 May) to 28.5% on the last sowing date (10 June) (Figure 1B). In contrast, soil pH varied within a range of 8.52 to 8.68, without a consistent variation as observed for temperature and moisture (Figure 1C).

Based on these findings, we analyzed the correlations between the soil parameters and disease indices (Figure 1D). Among the soil factors evaluated, soil temperature demonstrated the strongest correlation with disease severity (correlation coefficient r1 = −0.98, *p* = 3.4 × 10^−3^), followed closely by soil moisture (correlation coefficient r2 = 0.96, *p* = 9.7 × 10^−3^). Additionally, a significant negative correlation was observed between soil temperature and moisture (correlation coefficient r3 = −0.97, *p* = 7.4 × 10^−3^). In contrast, soil pH was not correlated with disease severity (correlation coefficient r4 = −0.28, *p* = 0.64). These results suggest that with delayed sowing, fluctuations in air temperature influenced soil temperature, which in turn led to the variation of soil moisture.

### 3.4. The Variation of Microsclerotia in Rhizosphere Soil of Sunflower with Delayed Sowing

**Figure 2:**
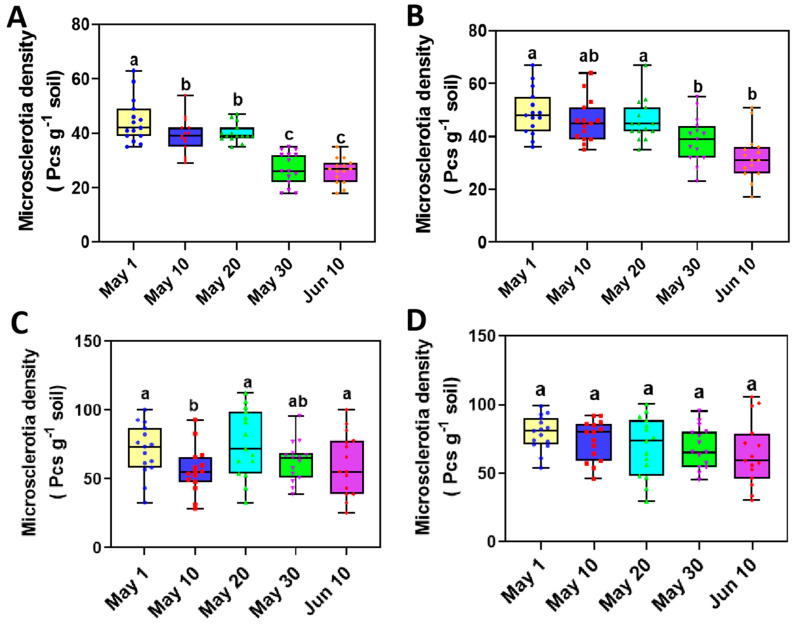
Correlations between microsclerotia number in rhizosphere soil and different sowing dates of sunflowers. (**A**) Number of microsclerotia in the rhizosphere soil when sunflowers reached V2 (fourth leaf) growth stage at different sowing date plots. (**B**) Number of microsclerotia in the rhizosphere soil when sunflowers reached V6 (eighth leaf) stage at different sowing date plots. (**C**) Number of microsclerotia in the rhizosphere soil when sunflowers reached R1 (early budding) stage at different sowing date plots. (**D**) Number of microsclerotia in rhizosphere soil when sunflowers reached R5 (mid-blooming) stage at different sowing date plots. Box plot illustrates the distribution of data, with the median represented by the central line in each box. The interquartile range (IQR), spanning from the first quartile (Q1) to the third quartile (Q3), captures the central 50% of values. Whiskers extend to smallest and largest values within 1.5 times the IQR from Q1 and Q3. Data points outside this range are displayed as outlier plots showing median and interquartile range of each treatment group. Statistical differences were analyzed using one-way ANOVA, followed by Tukey’s HSD test (*p* < 0.05). Different letters mean significant differences among groups (*p* < 0.05).

The amount of microsclerotia in the rhizosphere soil of sunflowers planted in different sowing date plots was assessed using NP-10 selection medium across different developmental stages. The results showed a decreased tendency in the total amount of microsclerotia in the rhizosphere soil at both the V2 and V6 stage with delayed sowing (Figure 2). At the V2 stage, the average number of microsclerotia in soil from the first sowing date (1 May) was 44.53 per gram of soil, which was significantly higher than that 29.27 per gram of soil of the last sowing date (10 June). A similar trend was also observed at the V6 stage, where the number of microsclerotia in soil from the first sowing date was 49.73 per gram of soil, whereas, it is 35.73 per gram of soil from the last sowing date. However, no significant difference in microsclerotia numbers was observed in soil collected from the fields during the R1 and R5 stages.

**Figure 3:**
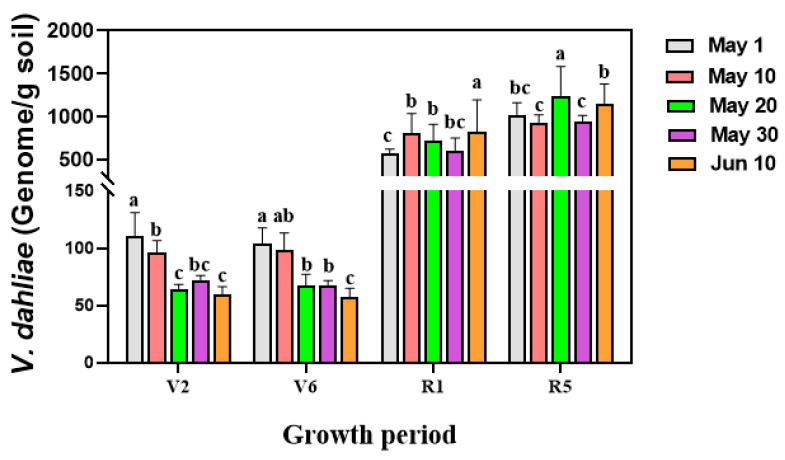
Quantification of biomass of *V. dahliae* in rhizosphere soil using qRT-PCR at different developmental stages at location 3. (i) Different growth stages of sunflower: V2 stage = fourth leaf stage; V6 stage = eighth leaf stage; R1 stage = early budding period; R5 stage = mid-blooming period. Treatment means were compared using one-way ANOVA, followed by Tukey’s HSD test (*p* < 0.05). All statistical analyses were performed in SPSS (version 22.0, IBM Corp., Armonk, NY, USA). Different letters denote significant differences among treatments (*p* < 0.05).

The total amount of microsclerotia in the soil was also quantified using qRT-PCR with DNA isolated from soil samples collected from different sowing dates. This analysis also confirmed a decreasing trend of the pathogen in soil with delayed sowing, consistent with the observed reduction of microsclerotia amount in soil (Figure 3). Specifically, at the V2 stage, the average amount of pathogen in soil of the first sowing date (1 May) was 111.16 genome per gram of soil. In contrast, it was 63.38 genome copies per gram of soil at the last sowing date (10 June). This significant reduction in the pathogen biomass indicates that delayed sowing leads to a decrease in the amount of *V. dahliae* in rhizosphere soil (Figure 2). This trend was consistent only across V2 and V6 seedling stages of sunflower.

### 3.5. Decrease in V. dahliae Colonization in Sunflower Roots with Delayed Sowing Dates

**Figure 4:**
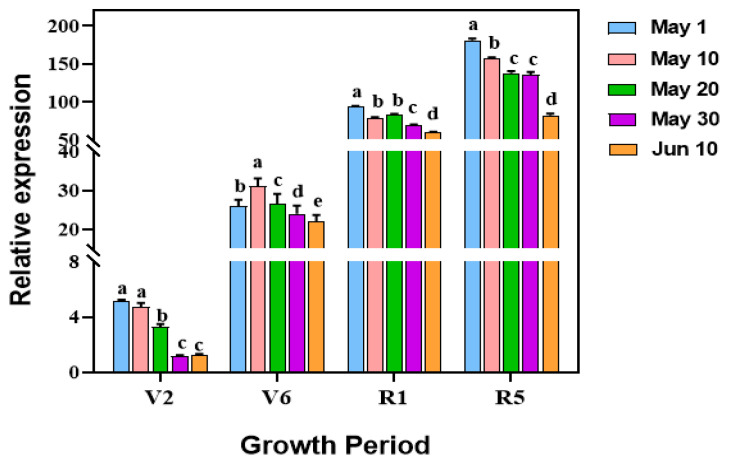
Quantification of the biomass of *V. dahliae* inside sunflower roots using qRT-PCR at different growth stages at location 3; V2 stage (fourth leaf stage), V6 stage (eighth leaf stage), R1 (early budding period) stage, and R5 (mid-blooming period). Treatment means were compared using one-way ANOVA, followed by Tukey’s HSD test (*p* < 0.05). Different letters denote significant differences among treatments (*p* < 0.05).

The biomass of *V. dahliae* colonized inside sunflower roots was also quantified using qRT-PCR at different sowing dates. The results showed the variability of biomass of *V. dahliae* colonized inside roots across different sowing dates. However, a clear decreasing trend was observed with delayed sowing dates at different developmental stages (Figure 4). At the V2 stage, the biomass of *V. dahliae* inside sunflower roots significantly decreased with delayed sowing. This reduction pattern persisted until the sunflower flowering stage, although the biomass of the pathogen gradually increased as the sunflower developed.

**Figure 5:**
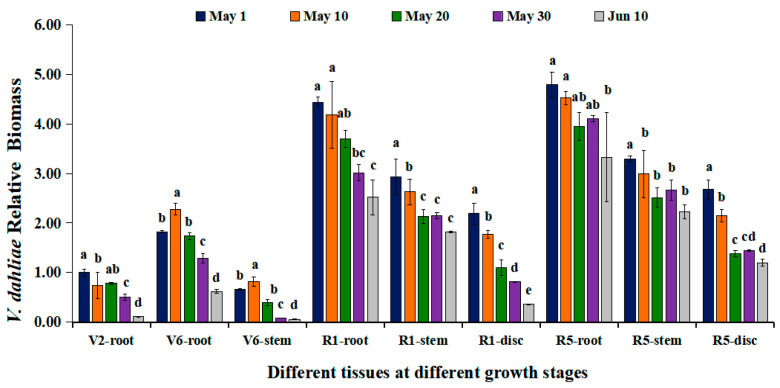
Quantification of *V. dahliae* biomass in the sunflower, detected by qRT-PCR in response to sunflower growth stages in a greenhouse. (i) V2 stage, fourth leaf stage; V6 stage, eighth leaf stage; R1 stage, early budding period; R5 stage, mid-blooming period; (ii) root, stem, and disc (capitula) represent plant sampling organs. Treatment means were compared using one-way ANOVA, followed by Tukey’s HSD test (*p* < 0.05). Different letters denote significant differences among treatments (*p* < 0.05).

To validate the results obtained from roots collected from different plots in the field, we conducted a parallel experiment in the greenhouse via artificial inoculation of *V. dahliae*. The variation of biomass of *V. dahliae* inside the sunflower roots collected from pots with different sowing dates mirrored the patterns observed in the field experiments (Figure 5). This confirms that delayed sowing could lead to a reduction in the total amount of pathogen in the soil, which in turn decreases pathogen colonization inside the roots. Findings from the pot experiments revealed that at four distinct growth stages of sunflower (V2, V6, R1, and R5), pathogens colonized inside the roots of the last sowing date (10 June) were significantly lower than those observed in the first sowing date (1 May). Notably, quantification of the pathogen colonized inside different parts of sunflowers planted in pots revealed that *V. dahliae* could spread upward through the vascular system once it is successfully colonized inside the vascular of the roots.

### 3.6. Effects of Delayed Sowing on Microbial Compositions in Rhizosphere Soil of Sunflower

**Figure 6:**
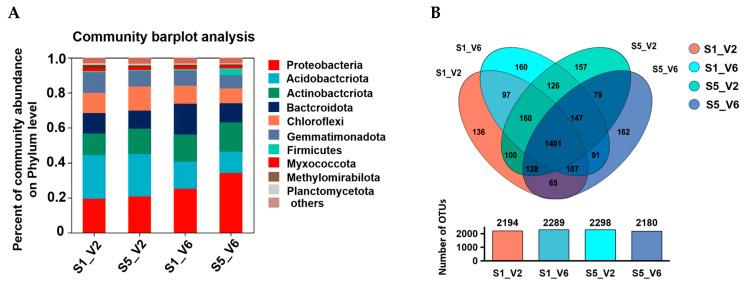
Analysis of species composition in different rhizosphere soil of sunflower under different sowing dates. (**A**). Community barplot analysis:Relative abundance of dominant bacteria at phylum classification level in different groups; (**B**). Venn focused analysis: number of unique and shared species in different groups. Species were determined based on operational taxonomy unit (out) levels.

To unravel whether delayed sowing affects the microbial composition in sunflower rhizosphere soils, high-throughput sequencing of the 16S rRNA gene was performed to characterize the bacterial community in soil samples collected from different sowing date plots. A total of 1,253,147 high-quality rRNA sequences were obtained from 27 soil samples, with an average length of 419 bp. Of these, 96.38% of the bacterial sequences were affiliated with 41 phyla, 113 classes, 229 orders, and 463 families. The relative abundance of dominant bacterial phyla at each classification level was visualized using a bar chart (Figure 6). The bacterial composition in rhizosphere soil varied across different sowing dates, with common dominant bacterial phyla (relative abundance > 1%) including *Proteobacteria*, *Acidobacteriota*, *Actinobacteriota*, *Bacteroidota*, *Chloroflexi*, *Gemmatimonadota*, *Firmicutes*, *Myxococcota*, *Methylomirabilota*, and *Planctomycetota,* which were identified in all soil samples. However, the relative abundance of these phyla differed among collected soil samples.

At the V2 stage, *Gemmatimonadota* and *Bacteroidota* were more abundant in soil samples collected from the first sowing date (1 May) compared to those from the last sowing date (10 June). Conversely, *Actinobacteria* and *Chloroflexi* were less abundant in the soil from the first sowing date. At the V6 stage, the populations of *Proteobacteria* and *Firmicutes* increased significantly with delayed sowing, while *Acidobacteriota*, *Bacteroidota,* and *Chloroflexi* showed decreased tendency.

Venn diagrams were used to illustrate the overlap and uniqueness of bacterial species (OTUs) among the different soil samples. These diagrams provided a clear representation of bacterial diversity and similarity (Figure 6B). The highest number of unique OTUs (162) was found in soil samples collected at the V6 stage of the last sowing date (10 June), followed by soil samples from the first sowing date (1 May) at the same developmental stage. At the V2 stage, the unique OUTs in soil from the last sowing date were also higher compared to those from the first sowing date. The percentage of specific OTUs in various sowing date–developmental stage combinations, such as S1-V2, S1-V6, S5-V2, and S5-V6 (Table 2), accounted for 6.19%, 6.98%, 6.83%, and 7.43% difference in total OTUs, respectively.

### 3.7. Beta Diversity

**Figure 7:**
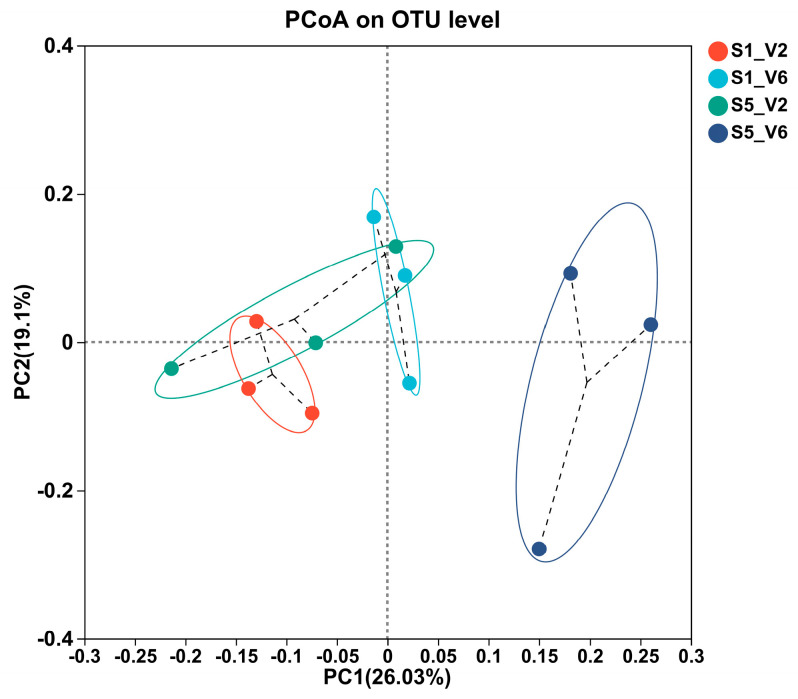
Principal coordinates analysis (PCoA) of sunflower rhizosphere soil under different sowing dates analyzed based on Bray–Curtis distance at the operational taxonomy unit (out) level in the field.

Principal Coordinates Analysis (PCoA) was used to visualize the similarities and differences in bacterial community composition among soil samples collected under different sowing dates and sunflower developmental stages. The analysis revealed that all collected soil samples could be grouped into four distinct clusters based on these parameters (Figure 7). The PCoA results showed that the groups corresponding to the last sowing date (10 June, S5) at the V6 stage exhibited considerable heterogeneity and did not overlap with any of the other three groups. In contrast, there was some overlap between the PCoA group for S5-V2 and those for S1-V2 and S1-V6, indicating similarities in bacterial community composition across these groups. PERMANOVA analysis confirmed that the differences in bacterial communities among the different combinations of sowing dates and sunflower developmental stages were statistically significant, under a *p*-value of 0.001 level. This result underscores the impact of the sowing date and developmental stage on the bacterial community structure in the rhizosphere soil of sunflowers.

**Figure 8:**
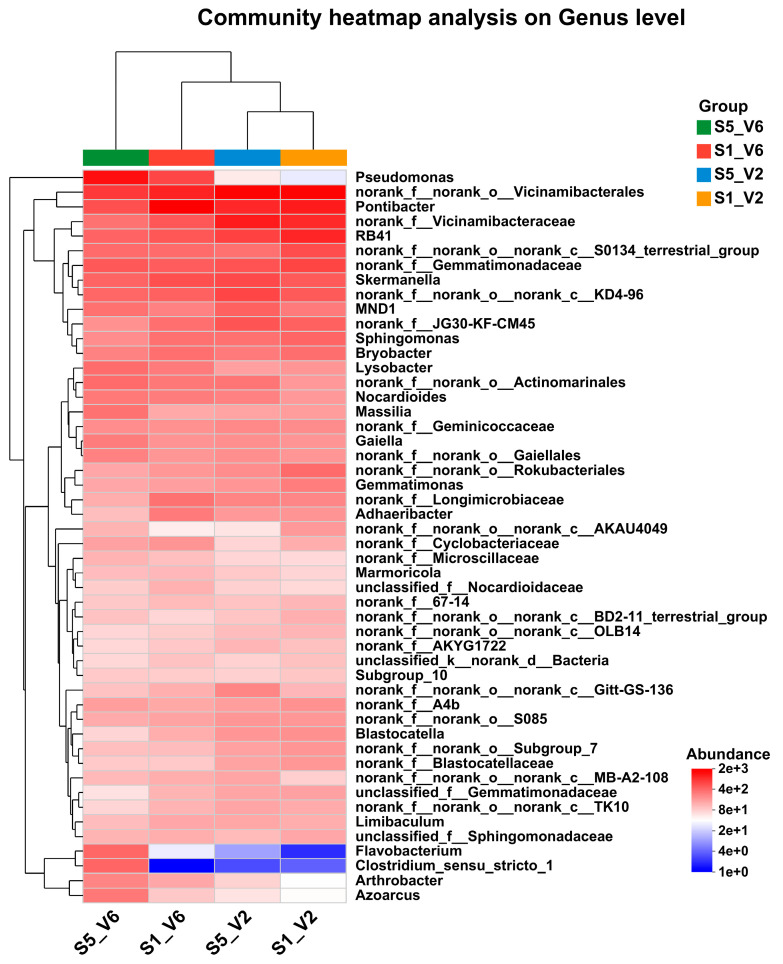
Community analysis at the genus level of sunflower rhizosphere soils under different sowing dates in the field.

A community heat map was used to visualize the distribution of the top dominant bacterial species across all tested soil samples, allowing us to explore shifts in dominant species among different treatments. The heat map comparison across various soil samples provided insights into the target microorganisms and their relative abundance. The results revealed that the abundance of certain bacterial genera, such as *Pseudomonas*, *Flavobacterium*, and *Azoarcus*, increased with delayed sowing date. Conversely, genera like *Pontibacter* and *Blastocatella* showed a decreasing trend (Figure 8). While many bacterial genera displayed a gradual shift in abundance, the changes were not as pronounced as those observed in the aforementioned genera.

### 3.8. Analysis of Species Variability

**Figure 9:**
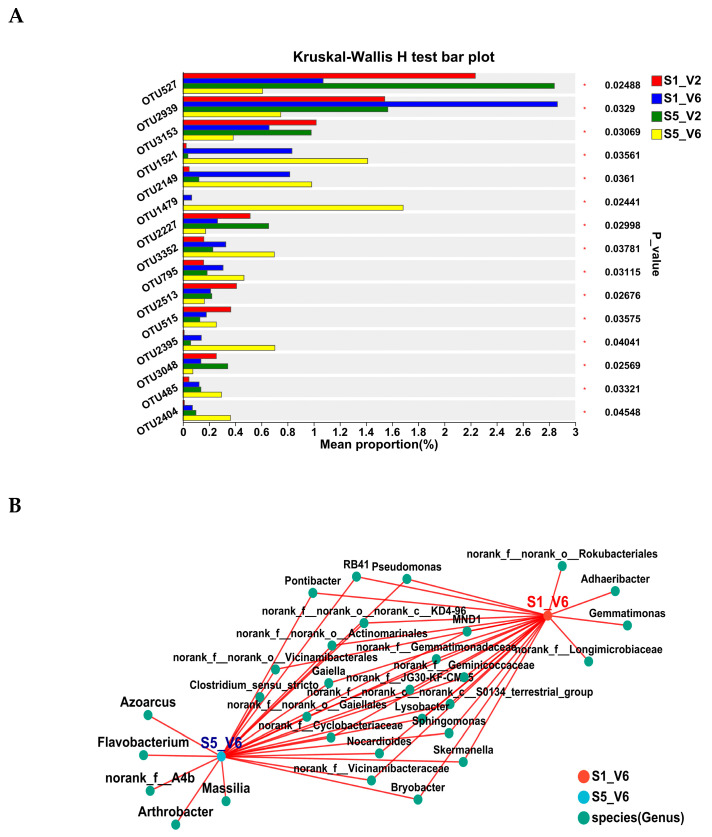
Bacterial species variability in sunflower rhizosphere soils across different combinations of sowing dates and growth stages. (**A**) Bar Plot of Multi-Species Differential Abundance Analysis. A bar chart was used to illustrate differences in the mean relative abundance of the same species across different groups, with significance levels (*p*-values) indicated by asterisks to denote statistically significant differences. This visualization provides a clear representation of the species’ significant differences among multiple groups. The Y-axis represents species names at a given taxonomic level, while the X-axis shows the mean relative abundance of the species across different groups. Bars of different colors correspond to distinct groups. The rightmost section displays the P-values, with significance levels indicated as follows: * 0.01 < *p* ≤ 0.05. (**B**) Co-occurrence Network of Microbial Taxa Associated with Different Soil Samples. The co-occurrence network illustrates the relationships between microbial taxa at the genus level (green nodes) in two soil samples, S1_V6 (red node) and S5_V6 (blue node). Red lines indicate significant co-occurrence interactions. The size and connectivity of each node reflect the number of associations with other taxa. The network highlights the shared and unique microbial genera associated with each sample.

The analysis of bacterial species across samples of different combinations was performed based on community abundance data. Focusing on the top 15 OTUs with the greatest variation in abundance, some OTUs, including OTU1521 (*Pseudomonas*), OTU2149 (*Pseudomonas*), OTU1479 (*Flavobacterium*), OTU3352 (*Azoarcus*), OTU795 (*Bacillus*), 0TU2395 (*Azoarcus*), OTU485 (*Streptomyces*), and OTU2404 (*Bacillus*), were significantly more abundant in rhizosphere soil samples from the V2-S5 stage compared to samples from both V2-S1 and V6-S1 stages (Figure 9A). These OTUs are associated with genera known for their potential antagonistic roles against various pathogens.

Combining Venn diagrams and co-occurrence network analysis (Figure 6B) revealed distinct co-occurrence relationships among bacterial species in the soil samples. This analysis showed significant differences in bacterial genera between samples. Specifically, soil samples collected at both V2 and V6 stages from the last sowing date (10 June, S5) exhibited more pronounced differences in microbial composition. Genera such as *Azoarcus*, *Arthrobacter*, *Flavobacterium*, and *Massilia* were more dominant in the V6-S5 soil samples.

Overall, the abundance of specific OTUs varied across soil samples collected from different sunflower developmental stages and sowing dates. Bacterial genera such as *Pseudomonas, Bacillus*, *Flavobacterium,* and *Azoarcus* were notably enriched in soil samples from the last sowing date (10 June, S5) plots (Figure 9B). These bacteria are commonly utilized as biocontrol agents to control different plant diseases [71,72].

### 3.9. Isolation and Identification of Bacterial Antagonist V. dahliae

**Figure 10:**
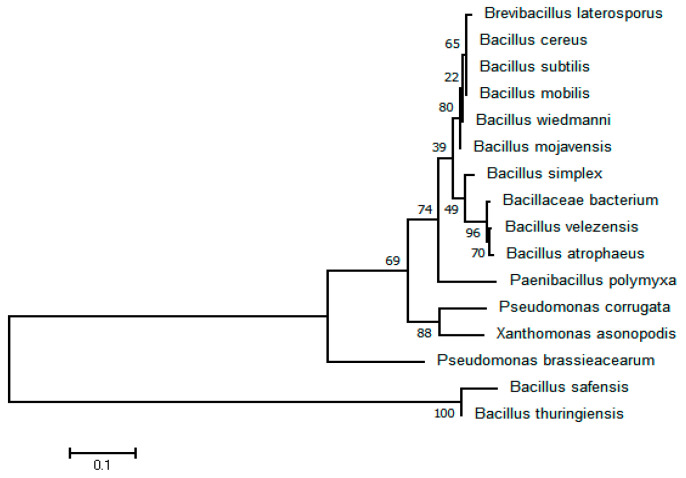
Phylogenetic tree of 16 isolated bacterial strains constructed based on 16S rDNA sequences.

To investigate the potential antagonistic functions of bacteria enriched in soil samples collected from the last sowing date plots (10 June, S5), we established a taxonomically diverse bacterial culture system. This system was used to isolate and identify bacterial species from the soil samples. Initially, we obtained a total of 7577 bacterial isolates using five different culture media (Supplementary Data S1). After eliminating duplicate colonies, 610 unique bacterial isolates were obtained. These isolates were taxonomically classified into 16 distinct bacterial species, and 16 bacterial isolates, including *Bacillus* spp., *Xanthomonas* spp., *Pseudomonas* spp., etc., were identified (Figure 10 and Supplementary Data S2).

### 3.10. Antagonist Function of Different SynComs on SVW

**Figure 11:**
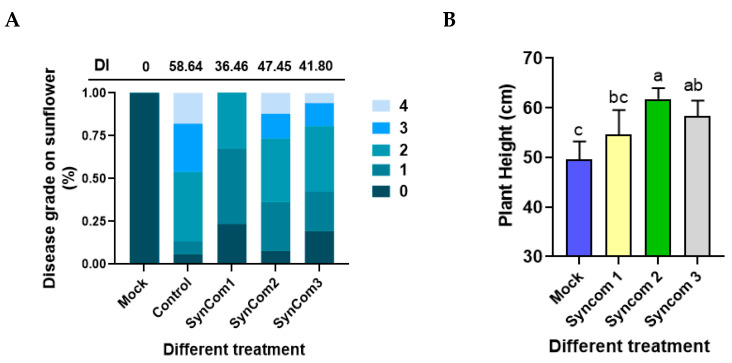
Effect of different SynComs treatments on the growth of sunflowers and disease index of SVW. (**A**) Statistical chart of disease grade of sunflower by different SynCom treatments. DI (Diseae Index); (**B**) Effect of different SynCom treatments on sunflower growth. Data were analyzed by one-way ANOVA, followed by Tukey’s HSD test for multiple comparisons (*p* < 0.05) using SPSS software (version 22.0). Error bars represent the standard deviation. Different lowercase letters indicate significant differences (Tukey’s multiple range test) (*p* < 0.05).

In a subsequent step, we evaluated the antagonistic function of the selected bacterial isolates using an artificial inoculation system under greenhouse conditions (Figure 11). We generated two different SynComs, which contain different species of bacteria (Table 3). SynCom1 contained five antagonistic strains of bacteria, and SynCom2 contained three strains of nitrogen-fixing bacteria. After the plant was pretreated with SynCom1, SynCom2, and SynCom3 (SynCom1 + SynCom2), compared with the control, the treatments of SynComs 1, 2, and 3 not only promoted the plant growth but also conferred substantially higher resistance against SVW (Figure 11B). This can be confirmed by the disease indices of SynCom1, SynCom2, and SynCom3 pretreatment, which are 36.46, 47.45, and 41.80 separately, whereas the disease index of the control plants is 58.64, thus the relative controlling effect is 37.82%, 19.08%, and 28.72%, respectively (Figure 11A). No additive control effects were observed in SynCom3, which mixed all the strains from both SynCom1 and SynCom2.

## 4. Discussion

In this study, we first determined that the delayed sowing date does reduce the severity of the SVW. We also observed that with the sowing date postponed, soil temperature increased, whereas soil moisture decreased correspondingly. These changes showed a significant correlation with the variation of disease severity, suggesting that disease occurrence is greatly influenced by soil environmental factors, especially for soilborne diseases. For soilborne diseases, the number of primary infection sources is a major determinant of disease severity [73]. Additionally, delayed sowing led to a reduction in the number of microsclerotia in the rhizosphere soil. This may be the reason for the decrease in the biomass of pathogens colonized inside the roots of sunflowers planted in the last sowing date plots (Figure 2 and Figure 3). This finding suggests that the initial density of pathogen in the soil could influence disease severity, and the pathogen suppression in the late sowing date plots may be due to the decreased amount of pathogens. Our results in this study also provide compelling evidence that the delayed sowing date of sunflowers can significantly reduce the incidence of SVW by promoting the abundance and activity of beneficial bacteria in rhizosphere soil (Figure 6, Figure 7 and Figure 8). The potential mechanism by which the delayed sowing date is affected is accompanied by soil temperature increasing gradually, and the quick development of the root system in the late sowing date facilitates the secretion of certain metabolite compounds, thus facilitating the attraction of certain species of bacteria. More beneficial microorganisms were attracted around sunflower roots in the late sowing date, promoting the growth of the plant and also enhancing the disease resistance to VW. Our findings support the hypothesis that sowing date plays a crucial role in shaping the rhizosphere microbial community, which in turn influences plant resistance levels.

In a previous study, the researchers found that adjustment of sowing dates was a method for disease control. For example, this approach has been applied to manage diseases such as northern corn leaf blight, powdery mildew, bean *Fusarium* root rot, lentil *Fusarium* wilt, and Groundnut rust [74,75,76,77]. *V. dahliae* produces microsclerotia that can persist in the soil for many years [1,78]. The density of microsclerotia is a crucial factor in predicting the severity of VW [79]. The dynamics of the total amount of microsclerotia in soil are influenced by the diseased plant debris in the soil and their mortality [16]. From an agronomic perspective, delayed sowing could be a valuable strategy to integrate into sunflower pest control strategy, particularly in areas prone to occur. This method offers a green agricultural approach to disease control.

Reduced Disease Incidence:

The observed reduction of disease severity of SVW after the delayed sowing date, suggests that soil environmental conditions, such as soil temperature and moisture at different sowing dates may be the driving force to affect both the pathogenicity of the pathogen and also the resistance level of the host (unpublished data). It has been shown that temperature and humidity changes affect the growth of the host, cell wall degrading enzyme activity, and infection structure development of pathogens, which eventually affect the pathogenicity [80,81,82,83]. For example, temperature changes significantly influence the speed of appressoria formation of rice blast fungus, *Magnaporthe oryzae* [84]. Also, soil temperature, moisture, and other conditions directly affect the viability of the pathogen, thus affecting its infestation ability [85]. Adjusting the sowing date leads to the variation in soil temperature and moisture, thus affecting the pathogen density and subsequently limiting the disease occurrence [86].

At present, studies have shown that temperature and humidity can regulate plant disease resistance by modulating the JA and SA signaling pathways [87,88]. However, little information is available regarding how temperature and humidity changes affect the pathogenicity of fungal pathogens in host plants. Based on our findings, delayed sowing of sunflowers appears to reduce the disease severity not only of SVW but also of Sunflower White Mold [89]. Similar effects were also observed on sunflower broomrape (*Orobanche cumana*) in the Inner Mongolia region [90]. These findings indicated that a delayed sowing date facilitates the changing of soil factors, thus affecting both the pathogen and also host. However, either soil temperature or humidity is the dominant factor, or both of them have additive effects on pathogens and the host is still elusive.

Enhancement of Beneficial Rhizosphere Bacteria:

The community structure of soil microorganisms serves as an important indicator of soil health and fertility. Differences in microbial communities between healthy and diseased plants can significantly impact the occurrence of soilborne diseases. For instance, in healthy wheat, beneficial microorganisms suppress pathogens, while in diseased plants, beneficial taxa diminish and pathogens proliferate dramatically [91]. These findings underscore the role of soil microbial affecting crop health, although the mechanism under such phenomena is still confusing.

The recruitment of the beneficial bacteria, particularly *Bacillus* and *Pseudomonas* species, to root rhizosphere, was observed in late-sown plants and is particularly noteworthy. These bacteria are known for their role in promoting plant growth and enhancing disease resistance via induced systemic resistance (ISR) and the production of antibiotics or enzymes that degrade pathogen cell walls [92,93,94,95,96]. Our results show that delayed sowing may create conditions favorable for the proliferation of these beneficial microorganisms, further contributing to the suppression of pathogen surrounding roots. Our data also suggest that the microbial community structure in the rhizosphere also changes obviously with a delayed sowing date, potentially due to soil temperature affecting the root exudates of sunflower plants, which can serve as carbon resources for microbes. Delayed sowing might also change the root exudate profiles, favoring the propagation of beneficial microbes over pathogens [97]. This could represent a novel approach to managing soilborne diseases by promoting the accumulation of beneficial microbes via delaying sowing dates.

In soil, the microbiome is the dominant factor to affects both pathogen population and pathogenicity and also promotes plant growth and induces systematic resistance of host plants. Thus, more research will be done to unravel the microbiome mobility accompanied by soil temperature variation, and this will give us some hints on the biological mechanism of delayed sowing date reduced disease severity of soilborne diseases.

The potential risk of this soilborne disease control technique:

Finally, we also need to consider the risks that arise from a delayed sowing date. One point we need to consider is the compatibility of the sunflower growth stage and frost-free period in the planting region. In principle, we do not recommend this agricultural technique in regions where the frost-free period is short. At least, when we plan to utilize delayed sowing to control soilborne disease, we need to premise the maturation of sunflower seeds, indicating this technique should be used regionally. Also, delayed sowing may also lead to the risk of new commercial pests in sunflower planting fields. For example, in Bayananoer, the sunflower main planting region in Inner Mongolia, with the sowing date delayed, we found that a big population of thrip (*Frankliniella intonsa*) moved from other crops to sunflower disks at the beginning of September. Large amounts of thrips gathered on the sunflower disk could cause damage to the sunflower husk, thus affecting its commercial value. Thus, we need to evaluate the potential risks of this technique in controlling soilborne diseases in the long term.

## Figures and Tables

**Table 1:** The information on the locations and sowing dates set up in this study between 2017 and 2019.

Location	Year	Sowing Dates	Sites	Coordinates	Sunflower Varieties and *Verticillium* Susceptibility ^z^	Previous Crop
1	2017	20 April, 1 May, 10 May, 20 May, 30 May	Minqin, WuWei, Gansu	N: 38°43′45″, E: 103°32′10″	LD5009 (S) T562 (R)	Sunflower
2	2018	1 May, 10 May, 20 May, 30 May, 10 June	Wuyuan, Inner Mongolia	N: 41°12′30″, E: 107°48′20″	LD5009 (S) KY-7 (R)	Sunflower
3	2019	1 May, 10 May, 20 May, 30 May, 10 June	Wulateqianqi, Inner Mongolia	N: 40°32′21″, E: 109°21′35″	LD5009 (S) JK601 (R)	Sunflower

^z^ R; *Verticillium* resistant; S: *Verticillium* susceptible.

**Table 2:** The combination of developmental stages and sowing date.

Number	Explanation
S1-V2	Sunflower V2 growth period, the first sowing date (S1)
S1-V6	Sunflower V6 growth period, the first sowing date (S1)
S5-V2	Sunflower V2 growth period, the fifth sowing date (S5)
S5-V6	Sunflower V6 growth period, the fifth sowing date (S5)

**Table 3:** SynCom compositions and bacterial strains.

Synthetic Composition	Strain Species	Strain Number
SynComs 1	*Bacillus thuringiensis*	A12-23
	*Bacillus atrophaeus*	T4-42
	*Pseudomonas brassieacearum*	G9-2
	*Bacillaceae bucterium*	A1-67
	*Bacillus cereus*	N3-16
SynComs 2	*Flavobacterium flevense*	S5-L-3
	*Arthrobacter gandavensis*	S5-B-1
	*Azoarcus* sp.	S5-A-1
SynComs 3	*Bacillus thuringiensis*	A12-23
	*Bacillus atrophaeus*	T4-42
	*Pseudomonas brassieacearum*	G9-2
	*Bacillaceae bucterium*	A1-67
	*Bacillus cereus*	N3-16
	*Flavobacterium flevense*	S5-L-3
	*Arthrobacter gandavensis*	S5-B-1
	*Azoarcus* sp.	S5-A-1

**Table 4:** Effects of delayed sowing dates on disease index of SVW at different locations.

Location	Sites	Sunflower Varieties	Average Disease Index of SVW at Different Sowing Dates					
			20 April	1 May	10 May	20 May	30 May	10 June
Field 1	Minqin, GS	LD5009(S)	55.01 a	46.04 b	34.38 c	25.59 d	23.02 d	——
		T562	27.86 a	19.12 b	22.57 b	14.79 bc	9.43 c	——
Field 2	Wuyuan, IM	LD5009(S)	——	66.56 a	57.75 ab	30.63 b	13.99 c	13.30 c
		KY-7(R)	——	39.87 a	29.36 b	19.94 c	5.94 d	6.96 d
Field 3	Wulateqianqi, IM	LD5009(S)	——	64.94 a	56.62 b	43.18 c	39.33 c	28.77 d
		JK601(R)	——	32.93 a	18.57 b	12.90 bc	15.09 bc	5.45 c
Pot experiment	Greenhouse, IMAU	LD5009(S)	——	54.15 a	56.94 a	45.69 b	28.16 c	15.43 d

Sunflowers were sown in different fields (Locations 1, 2, 3) during 2017–2019, the greenhouse experiment was performed in 2018. Sunflower varieties included the susceptible variety ‘LD5009’, and resistant varieties JK601(R), KY(R), and T562(R). “——” indicates the absence of planted crops, and therefore no data are available. Data were analyzed by one-way ANOVA, followed by Tukey’s HSD test for multiple comparisons (*p* < 0.05) using SPSS software (version 22.0). Different lowercase letters (a, b, c, and d) indicate significant differences (Duncan’s multiple range test) (*p* < 0.05).

**Table 5:** Sunflower subplot yield and thousand kernel weight (TKW) under different sowing dates.

Location	Sunflower Varieties		Subplot Yield and TKW of Sunflower at Different Sowing Dates					
			20 April	1 May	10 May	20 May	30 May	10 June
Field 1	LD5009(S)	Yield (Kg/P) ^z^	3.34 b	3.48 b	3.92 b	4.39 ab	4.67 a	——
		TKW (g)	80.44 b	88.96 ab	85.50 ab	97.06 a	96.78 a	——
	T562	Yield (Kg/P) ^z^	2.46 b	2.72 ab	3.63 a	3.68 a	3.87 a	——
		TKW (g)	51.80 b	56.24 ab	59.82 ab	68.98 a	69.76 a	——
Field 2	LD5009(S)	Yield (Kg/P) ^z^	——	4.41 c	4.87 bc	5.49 abc	6.31 ab	6.43 a
		TKW (g)	——	100.42 b	120.60 ab	123.24 ab	126.81 ab	140.88 a
	KY-7(R)	Yield (Kg/P) ^z^	——	7.58 b	8.73 a	8.31 ab	8.1 ab	7.74 ab
		TKW (g)	——	155.63 b	165.27 ab	169.01 ab	170.13 ab	189.45 a
Field 3	LD5009(S)	Yield (Kg/P) ^z^	——	3.76 b	3.84 b	4.38 b	4.93 ab	5.67 a
		TKW (g)	——	93.42 b	111.62 ab	115.05 a	117.67 a	120.43 a
	JK601(R)	Yield (Kg/P) ^z^	——	4.46 b	5.72 ab	6.63 a	6.68 a	6.87 a
		TKW (g)	——	103.04 b	126.42 ab	136.20 ab	140.88 a	146.21 a

^z^ Kg/P = Kg/subplot, 50 plants stand for each subplot yield. “——” indicates the absence of planted crops, and therefore no data are available. Different lowercase letters (a, b, and c) indicate significant differences (Duncan’s multiple range test) (*p* < 0.05).

## Data Availability

The original contributions presented in the study are included in the article/supplementary material, further inquiries can be directed to the corresponding author.

## Supplementary Materials

The following supporting information can be downloaded at: https://www.mdpi.com/article/10.3390/microorganisms12122416/s1, Data S1: Table of media used for bacteria isolation; Data S2: Details of Species Isolated from Rhizosphere Soil.
